# Conditional BDNF release under pathological conditions improves Huntington's disease pathology by delaying neuronal dysfunction

**DOI:** 10.1186/1750-1326-6-71

**Published:** 2011-10-10

**Authors:** Albert Giralt, Olga Carretón, Cristina Lao-Peregrin, Eduardo D Martín, Jordi Alberch

**Affiliations:** 1 Departament de Biologia Cel•lular, Immunologia i Neurociències, Facultat de Medicina, Universitat de Barcelona, Barcelona, Spain; 2 Institut d'Investigacions Biomèdiques August Pi i Sunyer (IDIBAPS), Casanova 143, Barcelona, Spain; 3 Centro de Investigación Biomédica en Red sobre Enfermedades Neurodegenerativas (CIBERNED), Spain; 4 Laboratory of Neurophysiology and Synaptic Plasticity, Albacete Science and Technology Park (PCYTA). Institute for Research in Neurological Disabilities (IDINE), University of Castilla-La Mancha, Albacete, Spain

**Keywords:** Huntingtin, synaptic plasticity, therapy, astrocytes, learning, Long-term potentiation

## Abstract

**Background:**

Brain-Derived Neurotrophic Factor (BDNF) is the main candidate for neuroprotective therapy for Huntington's disease (HD), but its conditional administration is one of its most challenging problems.

**Results:**

Here we used transgenic mice that over-express BDNF under the control of the Glial Fibrillary Acidic Protein (GFAP) promoter (pGFAP-BDNF mice) to test whether up-regulation and release of BDNF, dependent on astrogliosis, could be protective in HD. Thus, we cross-mated pGFAP-BDNF mice with R6/2 mice to generate a double-mutant mouse with mutant huntingtin protein and with a conditional over-expression of BDNF, only under pathological conditions. In these R6/2:pGFAP-BDNF animals, the decrease in striatal BDNF levels induced by mutant huntingtin was prevented in comparison to R6/2 animals at 12 weeks of age. The recovery of the neurotrophin levels in R6/2:pGFAP-BDNF mice correlated with an improvement in several motor coordination tasks and with a significant delay in anxiety and clasping alterations. Therefore, we next examined a possible improvement in cortico-striatal connectivity in R62:pGFAP-BDNF mice. Interestingly, we found that the over-expression of BDNF prevented the decrease of cortico-striatal presynaptic (VGLUT1) and postsynaptic (PSD-95) markers in the R6/2:pGFAP-BDNF striatum. Electrophysiological studies also showed that basal synaptic transmission and synaptic fatigue both improved in R6/2:pGAP-BDNF mice.

**Conclusions:**

These results indicate that the conditional administration of BDNF under the GFAP promoter could become a therapeutic strategy for HD due to its positive effects on synaptic plasticity.

## Background

Huntington's disease (HD) is an inherited neurodegenerative disorder, which results from the expansion of a CAG trinucleotide repeat in the *huntingtin *(htt) gene [1,2]. Mutant htt (mhtt) causes cortical atrophy and the preferential death of medium-sized spiny neurons in the striatum. The demise of these neurons causes motor and cognitive dysfunction [3]. In exon-1 and full-length mouse models of HD, a progressive decline in cortico-striatal synaptic function that correlates with the pathology has been described [4,5]. Thus, molecules regulating cortico-striatal connectivity would be extremely relevant to HD therapy perspectives.

Brain-Derived Neurotrophic Factor (BDNF) is an important neuroprotective factor for the preservation of medium-sized spiny neurons in the striatum [6]. However, the trophic support of striatal neurons is provided by cortical afferents [7,8]. A reduction in BDNF levels has been described in HD patients [9,10] and similar results have been found in several animal models [11-17]. Furthermore, the transport and function of BDNF are also disrupted in HD [18,19]. Thus, as one pathogenic mechanism leading to dysfunction and loss of striatal neurons in HD might be the reduction in BDNF levels, its administration should improve these alterations [20-22]. In fact, BDNF is a strong candidate for neuroprotective therapies, as it has been tested in acute [23,24] and in transgenic mouse models [12,25] of HD. However, the main challenge is to find the way to administer BDNF chronically and conditionally to the target neurons [22,26-28].

We previously demonstrated in an excitotoxic model of HD that transgenic astrocytes engineered to over-express BDNF under the control of the GFAP promoter, when grafted in wt mice, release higher levels of BDNF than control astrocytes [23]. This enhanced release of BDNF protects striatal neurons. Here we tested whether conditional and pathology-dependent delivery of BDNF could be neuroprotective in a transgenic mouse model of HD. We cross-mated R6/2 [29] with pGFAP-BDNF mice [23] to obtain double-mutant animals: R6/2:pGFAP-BDNF. Our results show that BDNF over-expression from striatal astrocytes improved HD phenotype by preventing cortico-striatal synaptic dysfunction.

## Results

### GFAP up-regulation increases BDNF levels in the striatum of R6/2:pGFAP-BDNF mice

In this study, we crossed mice that over-express BDNF under the control of the GFAP promoter (pGFAP-BDNF) [23] with the HD mouse model R6/2 [29]. With this procedure we obtained double-mutant R6/2:pGFAP-BDNF mice, which would express BDNF depending on the degree of astrogliosis.

**Figure 1:**
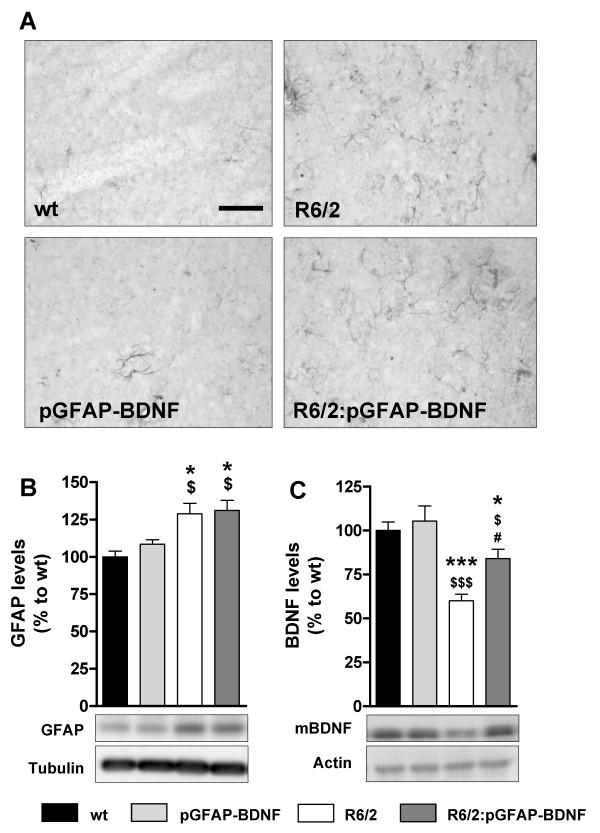
**Astrogliosis caused by the presence of mutant huntingtin induces preservation of striatal BDNF protein levels in R6/2:pGFAP-BDNF mice**. Striatal GFAP immuno-reactivity in 12-week-old wt, pGFAP-BDNF, R6/2 and R6/2:pGFAP-BDNF mice (A). The levels of GFAP (B) and mature BDNF (C) were analyzed by Western blot of protein extracts obtained from striata of 12-week old wt, pGFAP-BDNF, R6/2 and R6/2:pGFAP-BDNF mice. Representative immunoblots are also shown (B-C). Note loading control is different in each blot due to the huge difference on molecular weight of GFAP and mature BDNF and different blot characteristics. Values are expressed as mean ± s.e.m. Data were analyzed by one-way ANOVA: * *p *< 0.05 and *** *p *< 0.001, compared with the wt genotype; $ *p *< 0.05 and $$$ *p *< 0.001, compared with the pGFAP-BDNF genotype; and # *p *< 0.05, compared with the R6/2 genotype (n = 7-12/genotype). Scale bar 50 μm.

First, we checked whether 12-week-old R6/2 and R6/2:pGFAP-BDNF mice displayed astrogliosis by performing immunohistochemistry against GFAP. The results showed clear astroglial reactivity in the striatum of HD mouse models (Figure 1A). Accordingly, quantification by Western blot showed a significantly equal increase of striatal GFAP in R6/2 and R6/2:pGFAP-BDNF mice (Figure 1B). As previously observed in the excitotoxic model [23], this neuropathological hallmark induced an up-regulation of the pGFAP-BDNF transgene activity only in R6/2:pGFAP-BDNF mice, which correlates with significantly higher striatal BDNF levels than in R6/2 mice (Figure 1C). These results validated our model because although both, R6/2 and R6/2:pGFAP-BDNF mice displayed equal levels of striatal GFAP, the presence of the pGFAP-BDNF transgene only in the R6/2:pGFAP-BDNF mice prompted an increase and recovery of striatal BDNF levels in these mice relative to R6/2 mice who lacked the pGFAP-BDNF transgene.

### R6/2:pGFAP-BDNF mice show improvements in motor coordination

**Figure 2:**
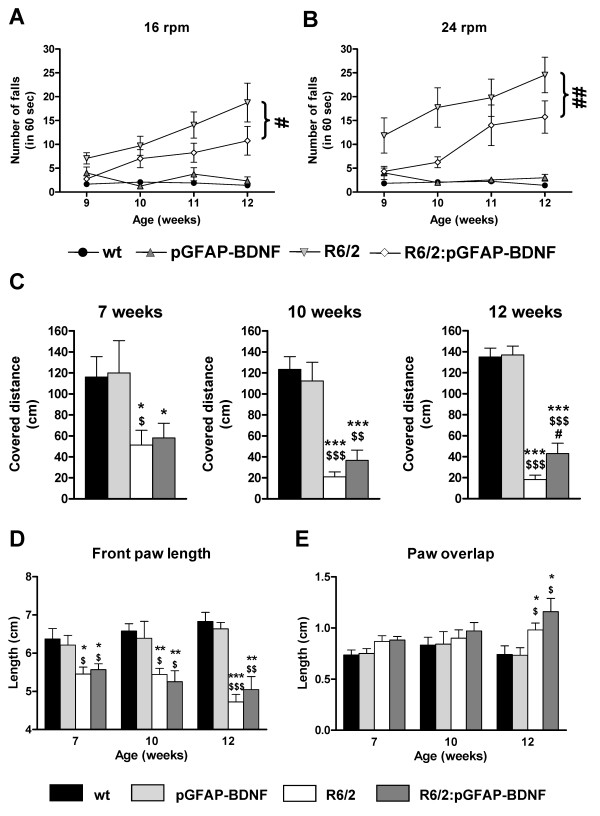
**Preservation of striatal BDNF levels in R6/2:pGFAP-BDNF mice is associated with an improvement of their motor performance over R6/2 mice**. Motor coordination and balance were analyzed by performing the rotarod task at 16 (A) and 24 (B) rpm in 9-, 10-, 11- and 12-week-old wt, pGFAP-BDNF, R6/2 and R6/2:pGFAP-BDNF mice. Motor coordination and balance were further assessed by the balance beam test at 7, 10 and 12 weeks of age (C). Ataxia and gait abnormalities were assessed by the footprint test, analyzing front paw length (D) and paw overlap (E) at 7, 10 and 12 weeks of age in wt, pGFAP-BDNF, R6/2 and R6/2:pGFAP-BDNF mice. Values are expressed as mean ± s.e.m. Data were analyzed by one-way ANOVA at different stages of the disease's progression in the case of the footprint and the balance beam tests because different cohorts of animals were used. In the case of the rotarod task, two-way ANOVA with repeated measures was performed. For clarity, in A and B only statistical comparisons between R6/2 and R6/2:pGFAP-BDNF mice are depicted. * *p *< 0.05, ** *p *< 0.01 and *** *p *< 0.001, as compared to wt mice; $ *p *< 0.05, $$ *p *< 0.01 and $$$ *p *< 0.001, as compared to pGFAP-BDNF mice; and # *p *< 0.05 and ## *p *< 0.01, as compared to R6/2 mice (n = 10-12/genotype).

We next evaluated motor coordination in wt, pGFAP-BDNF, R6/2 and R6/2:pGFAP-BDNF mice using the rotarod test. Disturbed motor coordination was observed in R6/2, compared to wt and pGFAP-BDNF mice at 9-10 weeks of age, at 16 rpm (Figure 2A) and 24 rpm (Figure 2B) and steadily worsened up to 12 weeks of age (Figure 2A-B). However, motor coordination deficits in R6/2:pGFAP-BDNF mice advanced with significantly less severity than in R6/2 mice (Figure 2A-B).

To further confirm better motor coordination in R6/2:pGFAP-BDNF than in R6/2 mice, we also evaluated their performance on the balance beam. The test with the balance beam was performed at 7, 10 and 12 weeks of age. The distance covered was the parameter used to check motor coordination. The results showed that performance was strongly affected in both R6/2 and R6/2:pGFAP-BDNF mice from 7 weeks of age (Figure 2C), before any overt phenotype is detectable. Interestingly, these alterations worsened in R6/2 but not in R6/2:pGFAP-BDNF mice, resulting in a significantly higher ability to cross the beam of R6/2:pGFAP-BDNF mice than of R6/2 mice at 12 weeks of age (Figure 2C).

**Figure 3:**
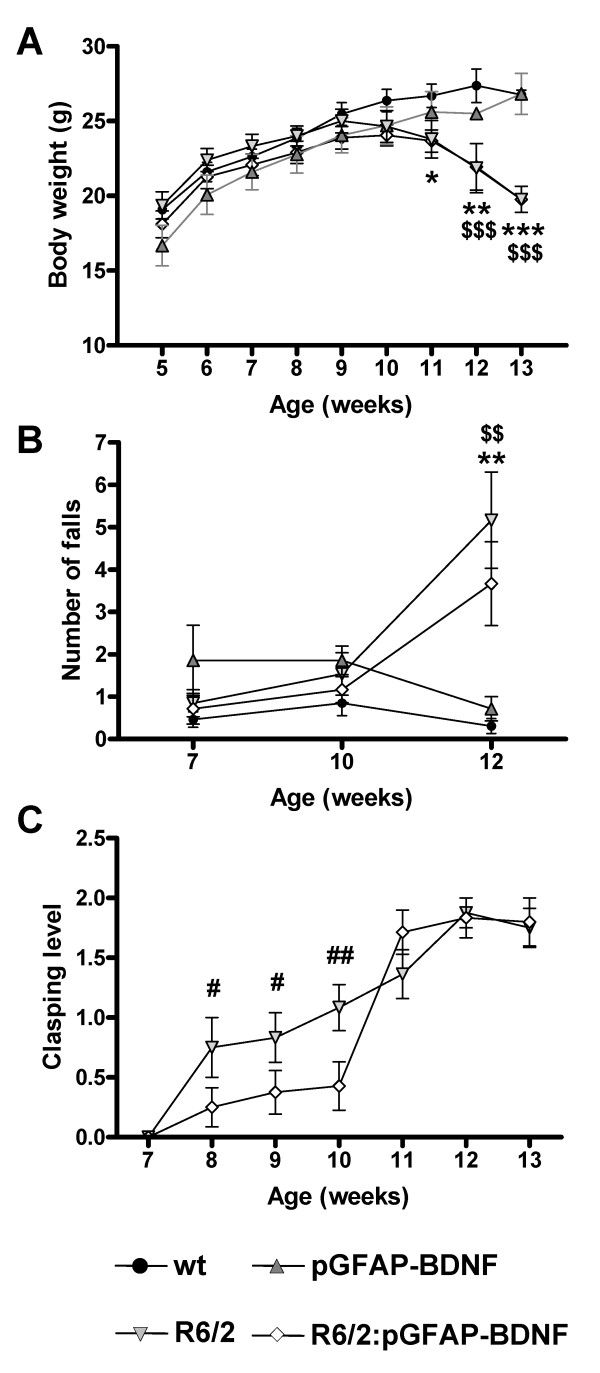
**The transgene pGFAP-BDNF delays clasping reflex alterations in R6/2 mice**. Body weights (A) of wt, pGFAP-BDNF, R6/2 and R6/2:pGFAP-BDNF mice were monitored weekly from 5 to 13 weeks of age. Muscular strength (B) was measured at 7, 10 and 12 weeks of age by the hiring wire test. Clasping level (C) was assessed weekly from 7 to 13 weeks of age. Values are expressed as mean ± s.e.m. Data were analyzed by two-way ANOVA with repeated measurements for body weight and muscular strength. Clasping level was measured by the *U *Mann-Whitney test. * *p *< 0.05, ** *p *< 0.01 and *** *p *< 0.001, as compared to wt mice; $ *p *< 0.05, $$ *p *< 0.01 and $$$ *p *< 0.001, as compared to pGFAP-BDNF mice; and # *p *< 0.05, as compared to R6/2:pGFAP-BDNF mice (n = 10-12/genotype).

Ataxia and gait abnormalities were evaluated by the footprint test. We found that both R6/2 and R6/2:pGFAP-BDNF mice displayed significant abnormalities in their front paw length (Figure 2D) and paw overlap (Figure 2E), when compared with their controls. Front paw length was significantly shorter in both R6/2 and R6/2:pGFAP-BDNF mice from 7 weeks of age, when loss of body weight was not yet evident (Figure 3A). No significant differences were observed between R6/2 and R6/2:pGFAP-BDNF mice (Figure 2D). On the other hand, paw overlap was significantly higher at 12 weeks of age in both R6/2 and R6/2:pGFAP-BDNF mice than in wt and pGFAP-BDNF mice (Figure 2E). In conclusion, the footprint test showed that gait and ataxia abnormalities were disturbed equally in R6/2 and R6/2:pGFAP-BDNF mice.

### The onset of the clasping reflex is ameliorated in R6/2:pGFAP-BDNF mice

Next, we studied additional phenotype alterations characteristic of R6/2 mice, such as loss of body weight and muscular strength and the development of the clasping reflex. Both R6/2 and R6/2:pGFAP-BDNF mice developed a normal body weight increase, compared with wt and pGFAP-BDNF mice, up to 10 weeks of age (Figure 3A). However, at this age R6/2 and R6/2:pGFAP-BDNF mice stopped gaining body weight; and at 11 weeks of age, both began to lose weight, with no significant differences between them (Figure 3A). Muscular strength was evaluated by the hiring wire test. This test showed that the muscular strength of R6/2 and R6/2:pGFAP-BDNF mice is normal, compared with wt and pGFAP-BDNF mice, up to 12 weeks of age, when both groups showed disorders in this parameter, as shown by a significant increase in falls from the grid (Figure 3B). On the other hand, both R6/2 and R6/2:pGFAP-BDNF mice steadily increased their clasping behavior (Figure 3C). However, R6/2 mice showed an early onset (8 weeks of age) of clasping behavior, whereas R6/2:pGFAP-BDNF mice started at 11 weeks of age (Figure 3C), suggesting significant delay of the associated neuronal dysfunction.

### Anxiety alterations are delayed in R6/2:pGFAP-BDNF mice

**Figure 4:**
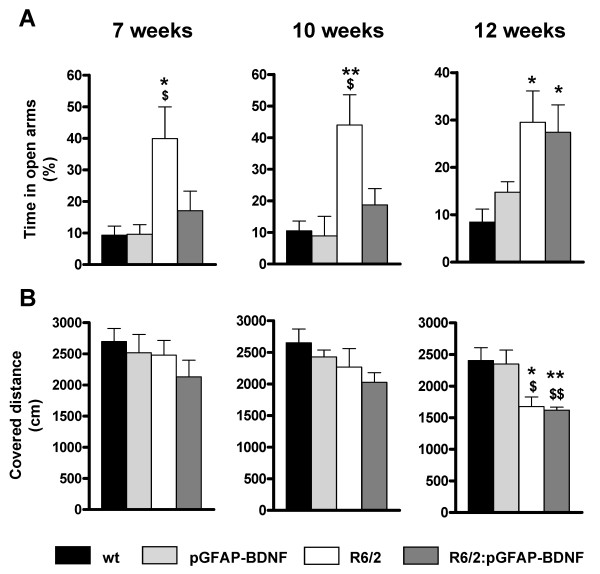
**The transgene pGFAP-BDNF delays the onset of anxiety disturbances in R6/2 mice**. The plus maze paradigm (A) was used to evaluate anxiety levels at 7, 10 and 12 weeks of age in wt, pGFAP-BDNF, R6/2 and R6/2:pGFAP-BDNF mice. The percentage of time spent on the open arms was monitored. The open field paradigm (B) was performed to check spontaneous locomotor and exploratory activities at 7, 10 and 12 weeks of age in wt, pGFAP-BDNF, R6/2 and R6/2:pGFAP-BDNF mice by recording the distance covered during 10 min. Values are expressed as mean ± s.e.m. Data were analyzed by one-way ANOVA because different cohorts of animals were used for the different stages of the disease's progression. * *p *< 0.05 and ** *p *< 0.01, as compared to wt mice; $ *p *< 0.05 and $$ *p *< 0.01, as compared to pGFAP-BDNF mice (n = 8-12/genotype).

To further characterize the behavioral phenotype improvements in R6/2:pGFAP-BDNF mice, compared to R6/2 mice, we also used the plus maze and the open field paradigms, which mainly check anxiety levels and spontaneous locomotor activity, respectively (see Methods). The results obtained in the plus maze indicated that at all ages analyzed (7, 10 and 12 weeks of age) R6/2 mice spent significantly more time in the open arms than wt and pGFAP-BDNF mice did (Figure 4A). This result suggests aberrant, decreased anxiety levels, as described elsewhere for these mice [30]. On the other hand, R6/2:pGFAP-BDNF mice showed a significant delay in the appearance of these alterations, which was not significant relative to wt mice until 12 weeks of age (Figure 4A). Thus, R6/2:pGFAP-BDNF mice showed an improvement in anxiety-like disorders.

To study the locomotor activity and exploration levels, we also performed the open field test. As previously described in HD mouse models [31,32], R6/2 and R6/2:pGFAP-BDNF mice showed a progressive decrease in spontaneous locomotor and exploratory activity in the open field, which reach significance relative to wt and pGFAP-BDNF mice at 12 weeks of age (Figure 4B). However, this significant trend to hypoactivity was equally developed in both R6/2 and R6/2:pGFAP-BDNF mice. These results suggest that less anxiety-like behavior in the plus maze shown by HD transgenic animals is unlikely to be caused by higher exploratory activity. Furthermore, the results indicate that the normalization of anxiety levels observed in R6/2:pGFAP-BDNF mice is not prompted by simple recovery of spontaneous locomotor activity.

### Loss of striatal volume and soma size of calbindin neurons and nuclear inclusions formation are ameliorated in R6/2:pGFAP-BDNF mice

**Figure 5:**
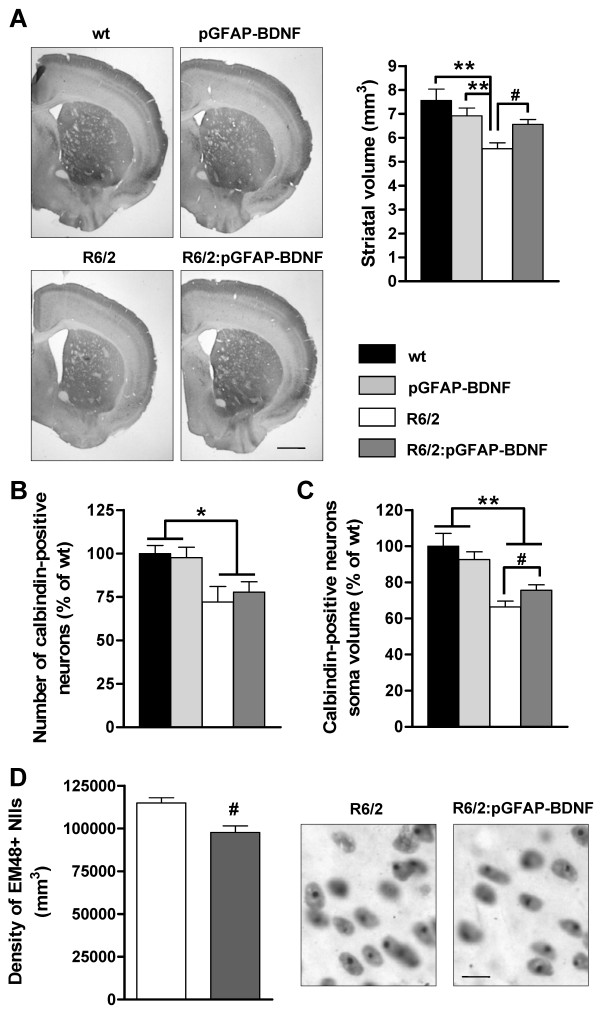
**Striatal neuropathology is improved in R6/2:pGFAP-BDNF mice compared to R6/2 mice**. Striatal volume (A) was stereologically measured in 12-week-old wt, pGFAP-BDNF, R6/2 and R6/2:pGFAP-BDNF mice. A representative microphotograph of coronal sections immunostained for calbindin is depicted (A). The number of calbindin-positive projecting neurons (B) was stereologically counted in all groups. The soma volume of calbindin-positive projecting neurons (C) was stereologically calculated in all genotypes by the nucleator method. Density of EM48-positive Nuclear Inclusions (NIIs) were stereologically counted in R6/2 and R6/2:pGFAP-BDNF mice (D). Values are expressed as mean ± s.e.m. Data were analyzed by one-way ANOVA or unpaired t-test. * *p *< 0.05 and ** *p *< 0.01, as compared to wt mice; # *p *< 0.05, as compared to R6/2 mice (n = 5-6/genotpye). Scale bar (A) 1 mm and (D) 10 μm.

As R6/2:pGFAP-BDNF mice showed greater preservation of their striatal-dependent motor coordination than R6/2 mice did, we next evaluated whether striatal neuropathology correlated with these behavioral results. First, although the pGFAP-BDNF transgene was expressed in the whole brain in R6/2:pGFAP-BDNF mice, this expression pattern was not enough to recover the reduction of total brain weight induced by mutant huntingtin (in grams; wt: 0.463 ± 0.013; pGFAP-BDNF: 0.455 ± 0.005; R6/2: 0.396 ± 0.014; R6/2:pGFAP-BDNF: 0.397 ± 0.020; *p *value < 0.05 for R6/2 and R6/2:pGFAP-BDNF mice compared with wt and pGFAP-BDNF mice). In contrast, stereological estimation of the striatal volume revealed that R6/2:pGFAP-BDNF mice have larger striata than R6/2 mice did (Figure 5A). Next we checked whether the striatal volume preservation found in R6/2:pGFAP-BDNF mice was due to greater survival of striatal projection neurons. Stereological counting of the calbindin-positive projection neurons in all groups indicated that both R6/2 and R6/2:pGFAP-BDNF mice have a similar significant decrease in the number of these neurons, in comparison with wt and pGFAP-BDNF mice (Figure 5B). However, when we measured the soma volume of calbindin-positive neurons, we found a significant volume improvement in R6/2:pGFAP-BDNF over R6/2 mice (Figure 5C). Finally, we wanted to check the number of Nuclear Inclusions (NIIs) in the striatum from our mouse models (Figure 5D). We observed that density of NIIs were lower in R6/2:pGFAP-BDNF relative to R6/2 mice striata (Figure 5D).

### Effect of BDNF over-expression on levels of DARPP-32 and enkephalin in the R6/2:pGFAP-BDNF striatum

**Figure 6:**
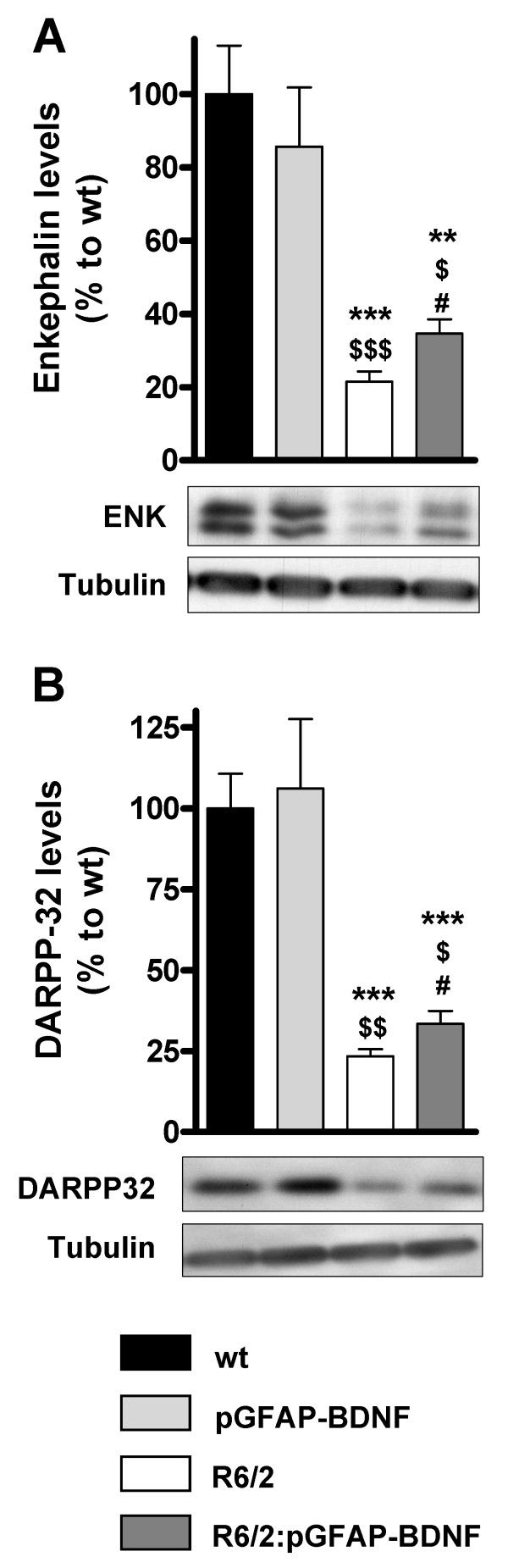
**The down-regulation of DARPP-32 and enkephalin protein levels in R6/2 mice is improved in R6/2:pGFAP-BDNF mice**. The levels of enkephalin (A) and DARPP-32 (B) were analyzed by Western blot of protein extracts obtained from striatum of 12-week-old wt, pGFAP-BDNF, R6/2 and R6/2:pGFAP-BDNF mice. Representative immunoblots are shown. Values are expressed as mean ± s.e.m. Data were analyzed by one-way ANOVA. ** *p *< 0.01 and *** *p *< 0.001, as compared to wt mice; $ *p *< 0.05, $$ *p *< 0.01 and $$$ *p *< 0.001, as compared to pGFAP-BDNF mice; and # *p *< 0.05, as compared to R6/2 mice (n = 7-12/genotype).

Enkephalin protein levels fall strongly in human HD brains and in mouse models [3,25,33,34]. Therefore, we used a specific antibody for Western blot that recognizes two enkephalin-positive bands at ~30 kDa [35] as previously described [36]. Our results show that R6/2:pGFAP-BDNF mice showed significant improvement in striatal enkephalin protein levels, compared with R6/2 mice (Figure 6A). To analyze the biochemical improvements in the striatum of R6/2:pGFAP-BDNF mice further, DARPP-32 protein levels were also analyzed in the striata from all genotypes (Figure 6B). DARPP-32 protein levels were significantly higher in R6/2:pGFAP-BDNF mice than in R6/2 ones (Figure 6B).

### Cortico-striatal synapses are preserved in R6/2:pGFAP-BDNF mice

As we found a preservation of the volume of striatal projection neurons in R6/2:pGFAP-BDNF mice, but not of their number, in comparison with R6/2 animals, we hypothesized that mainly subtle micro-structural changes were involved in R6/2:pGFAP-BDNF mouse phenotype improvements. Thus, we additionally tested the state of cortico-striatal connectivity by counting VGLUT1- and PSD-95-positive particle densities in the striatum of wt, pGFAP-BDNF, R6/2 and R6/2:pGFAP-BDNF 12-week-old mice.

**Figure 7:**
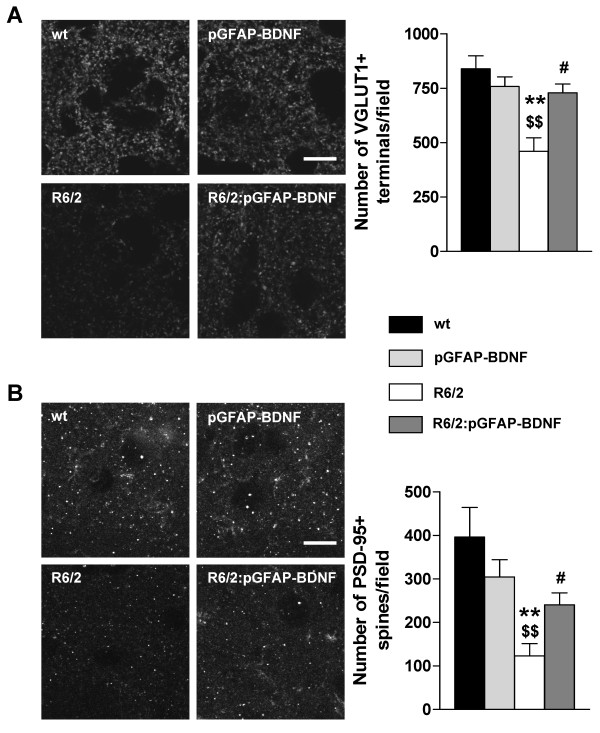
**Preservation of cortico-striatal connectivity in R6/2:pGFAP-BDNF mice**. Immunohistochemical staining against the pre-synaptic marker VGLUT1 (A) and the post-synaptic marker PSD-95 (B) was performed in 12-week-old wt, pGFAP-BDNF, R6/2 and R6/2:pGFAP-BDNF mice. The images were taken by confocal microscopy. VGLUT1- (A) and PSD-95- (B) positive particles were counted by the ImageJ software and represented in a graph as density of particles/field. Values are expressed as mean ± s.e.m. Data were analyzed by one-way ANOVA. * *p *< 0.05 and ** *p *< 0.01 as compared to wt mice; $ *p *< 0.05 and $$ *p *< 0.01 as compared to pGFAP-BDNF mice; # *p *< 0.05 as compared to R6/2 mice (n = 4/group). Scale bar 15 μm.

Confocal image analysis of coronal sections at 12 weeks of age showed that only R6/2 mice had a significant decrease in striatal VGLUT1- (Figure 7A) and PSD-95-positive (Figure 7B) particle density, in comparison with wt mice. However, R6/2:pGFAP-BDNF mice displayed highly intact striatal VGLUT1- and PSD-95-positive particle density, compared with wt mice (Figure 7). These results suggest that both excitatory terminals (VGLUT1) and dendritic spines (PSD-95) are preserved in R6/2:pGFAP-BDNF mice. Moreover, these results are in line with the volume preservation of the whole striatum and striatal calbindin-positive neurons in R6/2:pGFAP-BDNF mice, compared with R6/2 ones.

### Intact cortico-striatal synaptic transmission and resistance to synaptic fatigue in R6/2:pGFAP-BDNF mice

**Figure 8:**
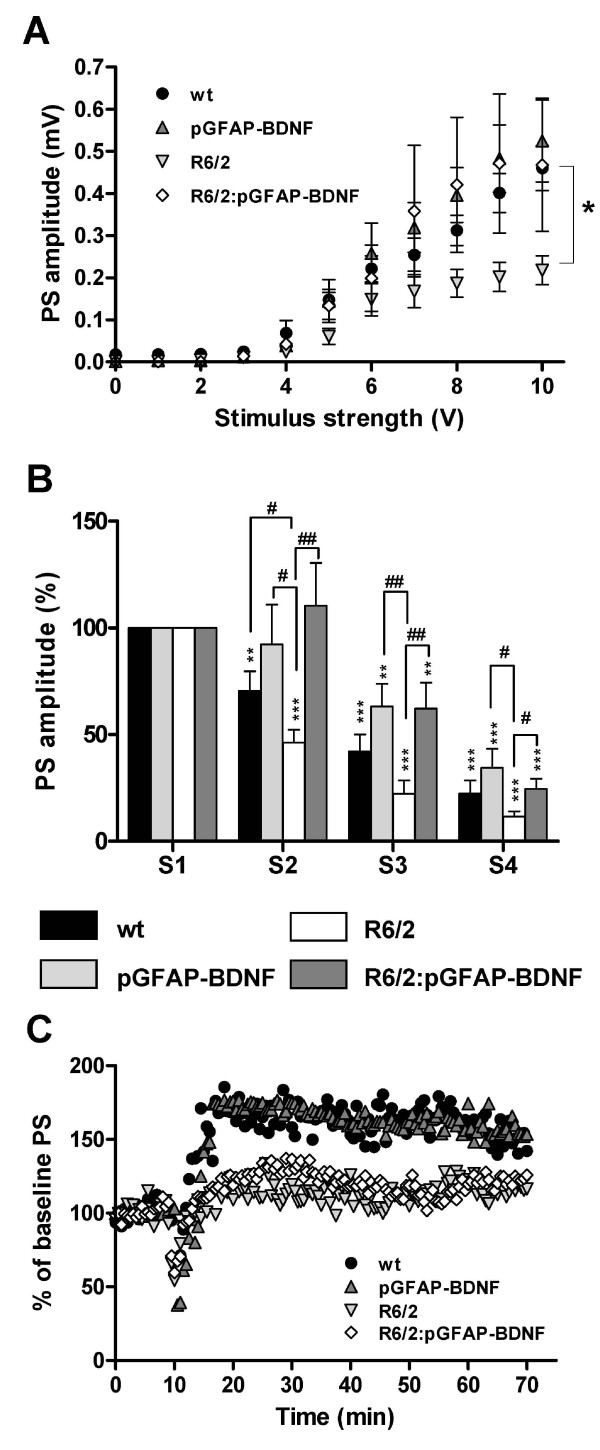
**The transgene pGFAP-BDNF counteracts synaptic fatigue in cortico-striatal connections**. Population spike (PS) amplitudes for a given range of stimulus intensities are shown from 12-week-old wt, pGFAP-BDNF, R6/2 and R6/2:pGFAP-BDNF mice (A). Synaptic fatigue using PS recordings was evoked by a four-stimulus train at 100 Hz (B). The PS response at the fourth initial stimulus was calculated as the percentage of the first PS amplitude. Long-term potentiation (LTP) was induced by high-frequency conditioning tetanus (C). Values are expressed as mean ± s.e.m except for C, where error bars are deleted for greater clarity. ** *p *< 0.01 and *** *p *< 0.001, for 100% control; # *p *< 0.05 and ## *p *< 0.01 for R6/2 mice (n = 8 slices/genotype (at least 3 animals in each case)).

Due to the improvement in the number of synaptic particles in R6/2:pGFAP-BDNF mice over R6/2 mice, we next examined glutamatergic synaptic transmission in the different groups. We made extracellular recordings of field potentials in acute cortico-striatal brain slices. As described in previous studies, single-pulse stimulation of striatal slices elicited a characteristic response with different components [37-39]. One of these components, the population spike (PS), reflects excitatory monosynaptic transmission in a population of striatal neurons due to endogenous glutamate release upon electrical stimulation of glutamate-containing fibers. This component is mediated by activation of α-amino-3-hydroxy-5-methyl-4-isoxazolepropionic acid (AMPA) glutamate receptors [38]. First, we analyzed basal synaptic transmission by applying isolated stimuli of increasing intensity. Extracellular field recordings showed that R6/2 mice tended to exhibit decreased PS responses, which were statistically significant at high stimulus strengths (Figure 8A; V = 10 V, *p *< 0.01). Interestingly, the amplitude of R6/2:pGFAP-BDNF PS responses was not significantly different from PS response in wt or pGFAP-BDNF mice (Figure 8A), indicating that the efficacy of excitatory synaptic inputs is greater in R6/2:pGFAP-BDNF mice. Since mhtt has been shown to impair synaptic transmission in the cortico-striatal pathway [40,41], we next examined synaptic fatigue by means of PS recordings evoked by a four-stimulus train (100 Hz). After the second stimulus in the train, the PS response was significantly smaller in R6/2 slices than in wt slices (Figure 8B, p < 0.05). Interestingly, the PS response in pGFAP-BDNF and R6/2:pGFAP-BDNF mice was significantly greater than in wt and R6/2 mice (Figure 8B), indicating that R6/2:pGFAP-BDNF mice were more resistant to synaptic fatigue.

We next investigated cortico-striatal synaptic plasticity by inducing LTP by high-frequency conditioning tetanus (see Methods). Baseline responses were monitored for 10-30 min before conditioning and were found to be stable. Tetanus conditioning revealed a marked difference in the ability of transgenic HD mice to support LTP, with strengthening of PS significantly low in R6/2 and R6/2:pGFAP-BDNF mice, whereas wt and pGFAP-BDNF mice had a significant PS response (Figure 8C, p < 0.001).

## Discussion

Here we demonstrate that conditional BDNF production and release from astrocytes is a safe and neuroprotective method to improve the R6/2 mice phenotype. Double-mutant R6/2:pGFAP-BDNF mice show improvements in striatal-dependent behavior, anxiety alterations and clasping levels, compared with R6/2 mice. In support of this, R6/2:pGFAP-BDNF mice show associated significant preservation of neuronal architecture, which correlates with improved cortico-striatal connectivity.

The neurotrophin BDNF has been widely put forward as a possible therapeutic molecule for HD treatment [20,22]. However, the key challenge in the field of growth factor therapy is drug delivery to the central nervous system. Another important consideration in the design of growth factor therapies for neurodegenerative disorders is the need for extended periods of treatment [42]. To date, there are no successful systems for delivering BDNF, because it does not cross the blood-brain barrier *via *peripheral administration [43,44]. On the other hand, approaches such as infection/lipotransfection and cell therapy are still inaccessible because of their collateral toxicity effects and the likelihood of their inducing aberrant proliferations, respectively. In addition, they are invasive procedures [27,45].

Both R6/2 mice [46-48] and HD human patients [49] show a striatal increase in astrogliosis reactivity with an associated up-regulation of GFAP levels. In the present study, we took advantage of striatal astrogliosis and the consequent up-regulation of GFAP promoter activity in R6/2 mice to produce and release BDNF physiologically. We accomplished this aim by crossing R6/2 mice with pGFAP-BDNF mice and obtaining double-mutant mice (R6/2:pGFAP-BDNF). The transgene works in a conditional fashion because it depends on the presence of astrogliosis, since pGFAP-BDNF mice did not show any increase in striatal BDNF levels compared with wt mice. Therefore, the increase in striatal BDNF in R6/2:pGFAP-BDNF mice, in comparison with R6/2 mice, was due to a conditional release, with auto-regulated mechanisms and only when it is pathologically required.

In this model, astrogliosis enables striatal BDNF expression to be significantly restored to close to control levels. Previous studies employed a *Bdnf *transgene driven by the promoter for the alpha subunit of Ca^2+^/calmodulin-dependent kinase II to over-express BDNF in the forebrain of R6/1 [12] and YAC128 [34] mice, showing high over-expression of BDNF that slowed the progression of the disease. Intriguingly, the beneficial effects observed in the present study are very similar to those described in these studies. Therefore, in an HD context, a small increase in BDNF levels is enough to provide a significant symptomatic improvement, which demonstrates the high relevance of this neurotrophin in the pathophysiology of the disease. This emphasizes the need to control the amount of BDNF delivered, since excessive chronic production and delivery of the neurotrophin to the brain may induce neuronal dysfunction and toxicity [50,51].

The behavioral characterization of R6/2:pGFAP-BDNF mice showed that the dysfunctions in motor coordination, evaluated by the rotarod and the balance beam tests, were delayed and improved, suggesting that the preservation of striatal BDNF levels had a strong positive effect on these mice. These results provide further support for the importance of BDNF in motor coordination function [52] and specifically of the striatum-dependent component [7]. Intriguingly, we did not find any changes in gait and locomotor deficits when mice performed the footprint and open field tests, which indicated that the improved striatal BDNF levels in R6/2:pGFAP-BDNF mice were not enough to ameliorate these symptoms. On the other hand, not only was motor coordination enhanced in R6/2:pGFAP-BDNF, but other neurological alterations typical of R6/2 mice were also improved, such as anxiety disturbances and the clasping reflex [29,30]. The improvement in anxiety alterations corroborated previous studies showing that one molecular mechanism able to modulate these processes was BDNF [53,54]. On the other hand, these results do not rule out possible functional improvements in other brain regions involved in the regulation of anxiety, such as the limbic-medial prefrontal circuit or the amygdala [55], in R6/2:pGFAP-BDNF mice and point to the need for further studies on this possibility.

Our data support the idea that neural dysfunction is a critical and early point in the pathophysiology of HD [56,57]. We found that the loss of soma volume in R6/2 projection neurons [40,58] improved in R6/2:pGFAP-BDNF mice. Interestingly, we also detected an amelioration of striatal NIIs density in R6/2:pGFAP-BDNF mice which is in accordance with their improved cellular phenotype. Moreover, the density of glutamatergic cortical terminals (VGLUT1-positive) and dendritic spines (PSD-95-positive) in the striatum were both down-regulated in R6/2 striata, but preserved in R6/2:pGFAP-BDNF striata. These positive changes in synaptic markers and neuronal soma volume correlated with an absence of basal synaptic transmission dysfunction and a greater resistance to synaptic fatigue in R6/2:pGFAP-BDNF mice than in R6/2 mice. Intriguingly, pGFAP-BDNF mice showed a significant increase in synaptic fatigue relative to wt mice. This result suggests a subtle increase on BDNF levels in those mice which we were unable to detect. On the other hand, our results corroborate previous studies showing that BDNF application increases glutamatergic currents [59] and the number of dendritic spines [15,60]. However, cortico-striatal LTP alterations did not improve in R6/2:pGFAP-BDNF mice. Although striatal BDNF levels were higher in R6/2:pGFAP-BDNF mice than in R6/2, they did not reach wt levels. This result could explain the lack of improvement in LTP expression in cortico-striatal slices from R6/2:pGFAP-BDNF mice. In fact, Simmons and others showed that there was a reversal of the impairment in hippocampal LTP in knock-in mice when levels of BDNF were completely recovered [61]. These findings confirm the need for complete integrity in the BDNF pathway in order to regulate cortico-striatal LTP [62].

The finding that BDNF released from transgenic astrocytes in R6/2:pGFAP-BDNF mice improves and regulates their striatal-dependent behavior and synaptic transmission corroborates the recent hypothesis that astrocytes are involved in neuro-plasticity phenomena such as the regulation and strengthening of neurosignals and connectivity of neuronal networks, some of which are likely to be involved in cognitive functions at least [63-65]. Thus, it is important to highlight that our results suggest that astrocytes could modulate some of these plasticity processes *via *production and delivery of BDNF, probably into the excitatory synapse. This is possible since astrocytes are capable, though at low levels, of producing and releasing BDNF *per se *[23,66,67] and send very fine projections to the excitatory synapses in order to strengthen them [68]. Thus, our results suggest a possible role for BDNF released from astrocytes in the regulation of basal synaptic transmission and synaptic fatigue in excitatory cortico-striatal synapses.

In the present study we wanted to administer BDNF non-invasively using a genetic approach. However, other non-invasively strategies have been reported to increase BDNF in the HD brains such as drug administration [61,69,70]. Thus, combination therapy with the pGFAP-BDNF construct and administration of a drug such as ampakines [69] could be crucial to fully recover the HD phenotype.

Finally, it should also be noted that other neurodegenerative diseases are associated with reactive astrogliosis and the consequent up-regulation of GFAP gene activity. Along these lines, pathologies such as Alzheimer's disease [71,72], Parkinson's disease [73,74] and amyotrophic lateral sclerosis [75] show increased astrogliosis reactivity with a consequent up-regulation of GFAP levels. It would be highly relevant to test whether the pGFAP-BDNF transgene might benefit these diseases. This statement takes relevance since BDNF delivery in a transgenic AD mouse model prevents loss of glutamatergic synapses and improves cognition [76]. In conclusion, conditional BDNF delivery regulated by the GFAP promoter in astrocytes could well be a therapeutic strategy for treatment of HD and other neurodegenerative diseases.

## Methods

### Animal handling and care

To obtain double-mutant animals (R6/2:pGFAP-BDNF mice) we cross-mated male R6/2 mice [29] with female pGFAP-BDNF mice [23]. Both R6/2 and pGFAP-BDNF mice were obtained from distinct colonies bred in a B6CBA background strain. Wild type (wt) littermate animals were used as the control group. Mice were housed together in numerical birth order in groups of mixed genotypes with access to food and water *ad libitum *in a colony room kept at constant temperature (19-22°C) and humidity (40-50%) on a 12 h light/dark cycle. All experiments used male mice, blind-coded for genotype. Data were recorded for analysis by microchip mouse number. All animal-related procedures were in accordance with the European Community guidelines for the care and use of laboratory animals (89/609//EEC) and were approved by the local animal care committee of the University of Barcelona (99/01) and Generalitat de Catalunya (99/1094).

### Rotarod

Motor coordination and balance were evaluated on the rotarod apparatus at distinct rotations per minute (rpm), as described elsewhere [25]. In brief, animals at 5 weeks of age were trained at constant speed (24 rpm) for 60 sec. We performed two trials per day for three consecutive days; and the latency to fall and the number of falls during 60 sec was recorded. No differences between groups were detected at this period. After training and starting at 7 weeks of age, animals were evaluated once a week at 16 and 24 rpm, and the number of falls in a total of 60 sec was recorded. The animals were put on the rotarod several times until the addition of the latency to fall off reached a total of 60 sec. The curves representing the behavioral pattern were compared and the percentage of motor coordination function impairment was calculated as described elsewhere [77].

### Balance beam

The motor coordination and balance of mice were also assessed by measuring their ability to traverse a narrow beam [25]. The beam consisted of a long steel cylinder (50 cm) with a 50 mm-square cross-section and a 15 mm-round diameter. The beam was placed horizontally, 50 cm above the bench surface, with each end mounted on a narrow support. Animals were allowed to walk for 1 min along the beam, while their latency to fall, number of falls and distance covered were measured.

### Footprint

The footprint test was performed as described elsewhere [25]. Mice were trained to walk in a corridor that was 50 cm long and 7 cm wide. The forefeet and hindfeet of the mice were painted with non-toxic red and blue ink, respectively, and then given one run. The footprint pattern was analyzed for the number of steps on the white paper, the stride length was measured as the average distance of forward movement between each stride, and the forebase and hindbase widths were measured as the perpendicular distance between the left and right footprints of a given step.

### Hanging wire

Neuromuscular abnormalities were analyzed by the hiring wire test as described elsewhere [25,78] in 7-, 10- and 12-week old wt, pGFAP-BDNF, R6/2 and R6/2:pGFAP-BDNF mice. A standard wire cage lid was used. To test balance and grip strength, mice were placed on top of a wire cage lid. The lid was shaken slightly to cause the mouse to grip the wires and then turned upside down for 60 sec. The number of falls of each mouse was recorded.

### Clasping

Clasping was measured by suspending mice from their tails at least 1 foot above a surface for 1 min. A clasping event was defined by the retraction of either or both hindlimbs into the body and toward the midline. Mice were scored according to the following criteria: 0 = no clasping, 1 = clasping 2 paws, and 2 = clasping all paws.

### Plus Maze

To analyze mouse anxiety, we used the plus maze paradigm. The apparatus used was similar to that described elsewhere [30]. Briefly, the raised plus maze was made of wood and consisted of two opposing 30 × 8 cm open arms, and two opposing 30 × 8 cm arms enclosed by 15 cm-high walls. The maze was raised 50 cm from the floor and lit by dim light. Each mouse was placed in the central square of the raised plus maze, facing an open arm and its behavior was scored for 5 min. At the end of each trial, any defecation was removed and the apparatus was wiped with 70% alcohol. We recorded the time spent in the open arms, which normally correlates with low levels of anxiety. Animals were tracked and recorded with SMART junior software (Panlab, Spain).

### Open Field

To check spontaneous locomotor activity we used the open field [79]. Briefly, the apparatus consisted of a white circular arena measuring 40 cm in diameter and 40 cm in height. Dimly light intensity was 40 lux throughout the arena. Animals were placed on the arena center and allowed to explore freely for 10 min. Spontaneous locomotor activity was measured. At the end of each trial, any defecation was removed and the apparatus was wiped with 70% ethanol. Animals were tracked and recorded with SMART junior software (Panlab, Spain).

### Immunohistochemistry

Animals were deeply anesthetized with pentobarbital (60 mg/kg) and intracardially perfused with a 4% paraformaldehyde solution in 0.1 M sodium phosphate, pH 7.2. Brains were removed and post-fixed for 2 h in the same solution, cryoprotected with 30% sucrose in phosphate buffered saline (PBS) with 0.02% sodium azide and frozen in dry-ice cooled isopentane. Serial coronal sections (40 μm) obtained with a cryostat were processed for free-floating immunohistochemistry.

The sections were washed three times in PBS, permeabilized and blocked for 15 min by shaking at room temperature with PBS containing 0.3% Triton X-100 and 3% normal goat serum (Pierce Biotechnology). After three washes, brain slices were incubated overnight by shaking at 4°C with the corresponding primary antibodies in PBS with 0.02% sodium azide buffer. Antibodies used were the anti-PSD-95 1:500 (Affinity BioReagents) and anti-VGLUT1 1:500 (Synaptic systems). After primary antibody incubation, slices were washed three times and then incubated for 2 h shaking at room temperature with subtype-specific fluorescent secondary antibodies: Cy3 goat anti-rabbit and Cy2 goat anti-mouse (both 1:100; from Jackson ImmunoResearch). No signal was detected in controls incubated in the absence of the primary antibody. Slight modifications were performed for VGLUT1 immunohistochemistry [80].

For diaminobenzidine immunohistochemistry experiments, endogenous peroxidases were blocked for 30-45 min in PBS containing 10% methanol and 3% H_2_O_2_. Then, non-specific protein interactions were blocked with normal serum or bovine serum albumin. Tissue was incubated overnight at 4°C with the following primary antibodies: anti-GFAP (1:500; Dako A/S), anti-calbindin (1:10000; Sigma Chemical Co.) and anti-EM48 (1:500; Chemicon). Sections were washed three times in PBS and incubated with a biotinylated secondary antibody (1:200; Pierce) at room temperature for 2 h. The immunohistochemical reaction was developed using the ABC kit (Pierce) and made visible with diaminobenzidine. No signal was detected in controls in which the primary antibodies had been omitted.

### Stereology

Striatal volume estimations were performed as described elsewhere [25]. Unbiased blinded to genotype counting for genotype and condition was performed with Computer-Assisted Stereology Toolbox (CAST) software (Olympus Danmark A/S). For estimating mean cellular/perikaryal volumes of neurons (so-called local volumes) with design-based stereology, the "nucleator" method was used, as described elsewhere [81]. To determine neuronal and NIIs sub-population densities (neurons or NIIs per mm^3^) in the striatum, we used the dissector counting procedure in coronal sections spaced 240 μm apart, as described elsewhere [82].

### Confocal analysis and dendritic spine-like structures counting

Fluorescently stained coronal sections were examined blinded to genotype by confocal microscopy, using a Leica TCS SL laser scanning confocal spectral microscope with argon and helium-neon lasers. Images were taken with a 63× numerical aperture lens with 4× digital zoom and standard (one Airy disc) pinhole. For each mouse, at least 4 slices of 40 μm containing striatal tissue were analyzed. Between 4 and 6 representative images, from dorsomedial striatum, were obtained from each slice. For each image, the entire three-dimensional stack of images was obtained by the use of the Z drive present in the Leica TCS SL microscope, and the size of the optical image was 0.5 μm, with a separation of 4 μm between each one. The number of dendritic spine-like structures was counted by the freeware NIH ImageJ version 1.33 by Wayne Rasband (National Institutes of Health, Bethesda, MD), similar to described elsewhere [15].

### Western blotting

Mice (12 weeks old; n: 7-14 per genotype) were deeply anesthetized in a CO2 chamber. The brain was quickly removed and the striatum was dissected, frozen in dry ice and stored at -80°C until use. Briefly, striatal tissue was sonicated in 250 Ml lysis buffer (PBS, 1% Nonidet P40, 0.1% SDS, 0.5% sodium deoxycholate, 1 mM PMSF, 10 mg/ml aprotinin, 1 mg/ml leupeptin, 2 mg/ml sodium orthovanadate) and centrifuged at 12,000 rpm for 20 min. Striatal proteins (15 μg) were analyzed by 7.5% SDS-polyacrylamide gel electrophoresis and transferred to Immobilon-P membranes (Millipore). Gel blots were blocked in TBS-T (150 mM NaCl, 20 mM Tris-HCl (pH 7.5), 0.05% Tween 20) with 5% non-fat dry milk and 5% bovine serum albumin. Immunoblots were probed with anti-DARPP-32 (1:1000; BD Biosciences), anti-GFAP (1:1000; Dako A/S), anti-BDNF (1:1000; Santa Cruz Biotechnology) and an affinity-purified rabbit anti-enkephalin applicable for Western blotting [35]. All blots were incubated with the primary antibody overnight at 4°C in PBS 0.02% sodium azide buffer with shaking. After several washes in TBS-T, blots were incubated with IgG HRP-conjugated anti-mouse or anti-rabbit antibodies (Promega Biotechnology) and developed using the ECL Western blotting analysis system (Santa Cruz). Incubation with a monoclonal anti-β-tubulin antibody (1:50,000; Sigma Chemical Co.) was used as a loading control.

### Electrophysiology

Transverse brain slices (450 μm thickness) were prepared from mice, using conventional methods [83], and incubated for more than 1 h at room temperature (21-24°C) in artificial cerebrospinal fluid (aCSF). The aCSF contained (in mM) 124 NaCl, 2.69 KCl, 1.25 KH_2_PO_4_, 2 MgSO_4_, 26 NaHCO_3_, 2 CaCl_2 _and 10 glucose, and was gassed with 95% O_2 _and 5% CO_2_. Brain slices containing both striatum and cortex were transferred to an immersion recording chamber and superfused (2.5 ml/min) with gassed aCSF warmed to 32-34°C. After 1 h of equilibration, extracellular field potentials were recorded in the dorsomedial striatum by a glass microelectrode filled with 1 M NaCl solution on stimulation of the white matter between the cortex and the striatum with a bipolar tungsten electrode *via *a 2100 isolated pulse stimulator (A-M Systems, Inc.). Long-term potentiation (LTP) was induced by applying four 1 s, 100 Hz trains delivered every 10 s, and potentiation was measured for 1 h after LTP induction at 0.1 Hz. For each experiment, population spike (PS) amplitude was expressed as a percentage of average pre-tetanus baseline amplitude values. Synaptic fatigue was induced by a train at 100 Hz. Data were filtered (highpass, 0.1 Hz; lowpass 3 kHz) and digitized using a PowerLab 4/26 acquisition system (AD Instruments). The software Scope (AD Instruments) was used to display PS and measurements of the amplitude of PS.

### Statistical analysis

All data are expressed as mean ± s.e.m. Different statistical analyses were performed as appropriate and are indicated in the figure legends. Values of *p *< 0.05 were considered statistically significant.

## Competing interests

The authors declare that they have no competing interests.

## Authors' contributions

AG and OC carried out the behavioral tests. AG performed morphological and biochemical studies. CLP and EDM carried out the electrophysiological experiments. JA designed the experiments and wrote the paper with assistance from all other authors. All authors read and approved the final manuscript.
